# Prognostic Value of CXCL12 in Non-Small Cell Lung Cancer Patients Undergoing Tumor Resection

**DOI:** 10.3390/ph16020255

**Published:** 2023-02-07

**Authors:** Yurie Kogue, Hiroyasu Kobayashi, Yutaka Nakamura, Takatsugu Takano, Chihiro Furuta, Osamu Kawano, Taro Yasuma, Tadashi Nishimura, Corina N. D’Alessandro-Gabazza, Hajime Fujimoto, Esteban C. Gabazza, Tetsu Kobayashi, Ichiro Fukai

**Affiliations:** 1 Department of Pulmonary Medicine, Suzuka Chuo General Hospital, 1275-53, Yasuzukacho, Suzuka 513-8630, Japan; 2 Department of Pulmonary and Critical Care Medicine, Graduate School of Medicine, Mie University Faculty, Edobashi, Tsu 514-8507, Japan; 3 Department of Pathology, Suzuka Chuo General Hospital, 1275-53, Yasuzukacho, Suzuka 513-8630, Japan; 4 Department of Pulmonary Surgery, Suzuka Chuo General Hospital, 1275-53, Yasuzukacho, Suzuka 513-8630, Japan; 5 Department of Immunology, Graduate School of Medicine, Mie University Faculty, Edobashi, Tsu 514-8507, Japan; 6 Department of Pulmonary Medicine, Mie Chuo Medical Center, Hisaimyojincho, Tsu 514-1101, Japan

**Keywords:** non-small cell lung cancer, CXCL12, CXCR4, adjuvant chemotherapy, prognosis, surgical treatment

## Abstract

Adjuvant chemotherapy is commonly indicated in lung cancer patients undergoing surgical therapy because tumor recurrence is frequent. A biomarker that can predict tumor recurrence in the postoperative period is currently unavailable. CXCR4 receptor and its ligand CXCL12 play important roles in metastasis. This study investigated the value of tumor CXCL12 expression to predict prognosis and indicate adjuvant chemotherapy in non-small cell lung cancer patients. This study enrolled 82 non-small cell lung cancer patients. The expression of CXCL12 was evaluated by immunohistochemistry. The degree of CXCL12 expression was assessed using the Allred score system. Among all subjects, the progression-free survival and overall survival were significantly prolonged in cancer patients with low tumor expression of CXCL12 compared to patients with high tumor expression. Multivariate analysis showed that the increased level of CXCL12 is a significant predictor of progression-free survival and overall survival in NSCLC patients. Among subjects with high tumor CXCL12 expression, progression-free survival and overall survival were significantly improved in patients treated with adjuvant chemotherapy compared to untreated patients. These results suggest the potential value of tumor CXCL12 expression as a marker to predict prognosis and to indicate adjuvant chemotherapy after surgical tumor resection in non-small cell lung cancer patients.

## 1. Introduction

Primary lung cancer is the leading cause of cancer-related death in many industrialized countries [1]. Recurrence of non-small cell lung cancer (NSCLC) is common even in cases diagnosed at early stages or undergoing complete tumor resection [1]. Indeed, the prognosis is not good even in patients that underwent complete resection [2]. The 5-year survival rates in patients at surgical stages IA1, IA2, IA3, IB, IIA, and IIB are 91.6%, 81.4%, 74.8%, 71.5%, 60.2%, and 58.1%, respectively [2]. These relatively low survival rates, despite the early clinical stage of the tumors, are due to the use of imaging techniques that cannot detect the presence of micrometastasis. Therefore, postoperative adjuvant chemotherapy is recommended in NSCLC patients even after apparent complete tumor resection [3,4]. For example, Japan tumor guidelines recommend using tegafur uracil for cases at postoperative pathological stages IA, IB, and IIA, and cisplatin-based intravenous chemotherapy for cases at stages II and IIIA [3,4]. However, postoperative adjuvant chemotherapy may be harmful to the patients. Therefore, a tumor marker is needed to detect the presence of a remnant tumor or micrometastasis after surgical tumor resection. Several clinical parameters have been reported as potential tumor markers, but the results remain unconvincing [5].

CXCL12 is a chemokine released by cancer-associated fibroblasts, tumors, and normal cells that promotes the motility and migration of cancer cells expressing its membrane receptor CXCR4 [6]. The CXCL12/CXCR4 axis plays a critical role in the pathogenesis of cancer metastasis [6]. A remnant group of CXCR4-expressing NSCLC cells after surgical resection of the primary tumor can migrate to lymph nodes, contralateral lung, liver, brain, and bone marrow that express high levels of CXCL12 to form new metastatic foci [7,8]. In addition, the CXCL12/CXCR4 axis may also promote cell proliferation, tumor growth, and angiogenesis [9]. High expression levels of CXCL12 and CXCR4 indicate poor prognosis in patients with NSCLC [9].

Many studies have previously demonstrated the prognostic significance of CXCR4 in NSCLC [10]. Therefore, in this study, we focused on CXCL12 and investigated whether the expression of CXCL12 by tumor cells and cancer-associated fibroblasts is associated with the prognosis of NSCLC patients undergoing complete tumor resection. In addition, the potential of tumor expression of CXCL12 as a biomarker for indicating adjuvant chemotherapy was also evaluated.

## 2. Results

### 2.1. Expression of CXCL12 in Cancer Cells and Cancer-Associated Fibroblasts

**Figure 1:**
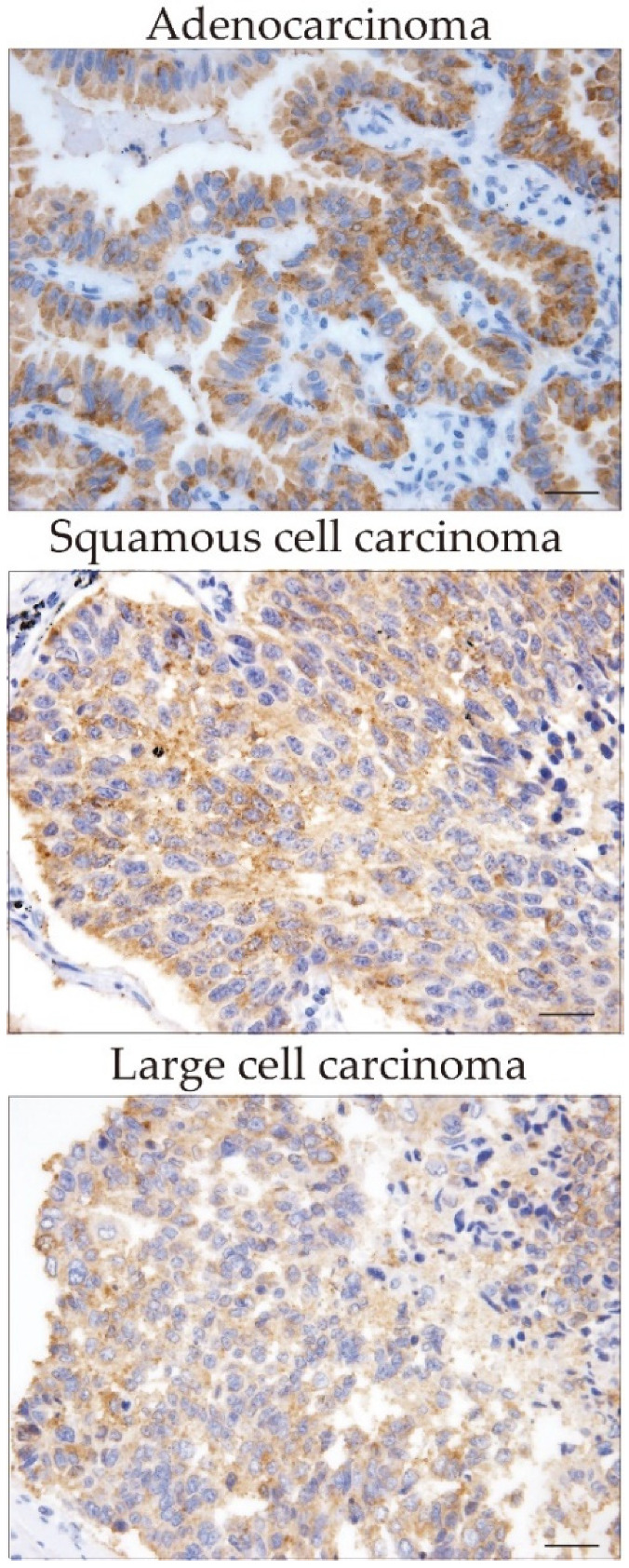
Expression of CXCL12 in tumor tissue from different histological types of non-small cell lung carcinoma. The expression of CXCL12 was evaluated as described under material and methods. Scale bars indicate 50 µm.

**Figure 2:**
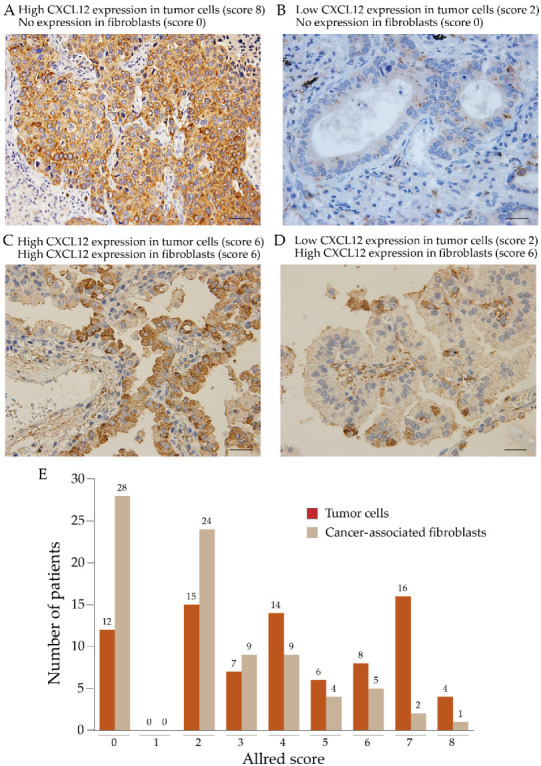
Expression of CXCL12 in tumor tissue from non-small cell lung carcinoma patients. The expression of CXCL12 was evaluated as described under material and methods. (**A**) Tumor with high expression of CXCL12 in tumor cells with no expression in cancer-associated fibroblasts. (**B**) Tumor with low expression of CXCL12 in tumor cells with no expression in fibroblasts. (**C**) Tumor with high expression of CXCL12 in tumor cells and cancer-associated fibroblasts. (**D**) Tumor with low expression of CXCL12 in tumor cells and increased expression in fibroblasts. (**E**) The number of patients in each Allred score. Scale bars indicate 50 µm (400× magnification). Overall, 85.3% (70/82) of tumor cells and 65.9% (54/82) of cancer-associated fibroblasts were positive for CXCL12.

Immunohistochemical analysis revealed the expression of CXCL12 in different tumor histological types (Figure 1) and variable degrees of CXCL12 expression in cancer cells and cancer-associated fibroblasts in lung cancer specimens (Figure 2A–E). Seventy patients (85.3%) showed positive expression of CXCL12 in tumor cells, and fifty-four patients (65.9%) showed positive expression of CXCL12 in cancer-associated fibroblasts. However, no correlation was found in the degree of CXCL12 expression between tumor cells and cancer-associated fibroblasts. Low expression of CXCL12 was observed in 39 patients, while high expression was observed in 43 patients (Table 1).

### 2.2. CXCL-12 Staining Was Not Associated with Clinicopathological Parameters

The relationship between clinical parameters and the sum of CXCL12 expression intensity in both cancer cells and cancer-associated fibroblasts was assessed. High CXCL12 expression was not significantly associated with age, gender, pathological stages, or lymphatic and blood vessel invasion. Most cases of squamous cell carcinoma showed low expression of CXCL12 (Table 1).

### 2.3. CXCL12 Expression Was Associated with the Prognosis of Cancer Patients

**Figure 3:**
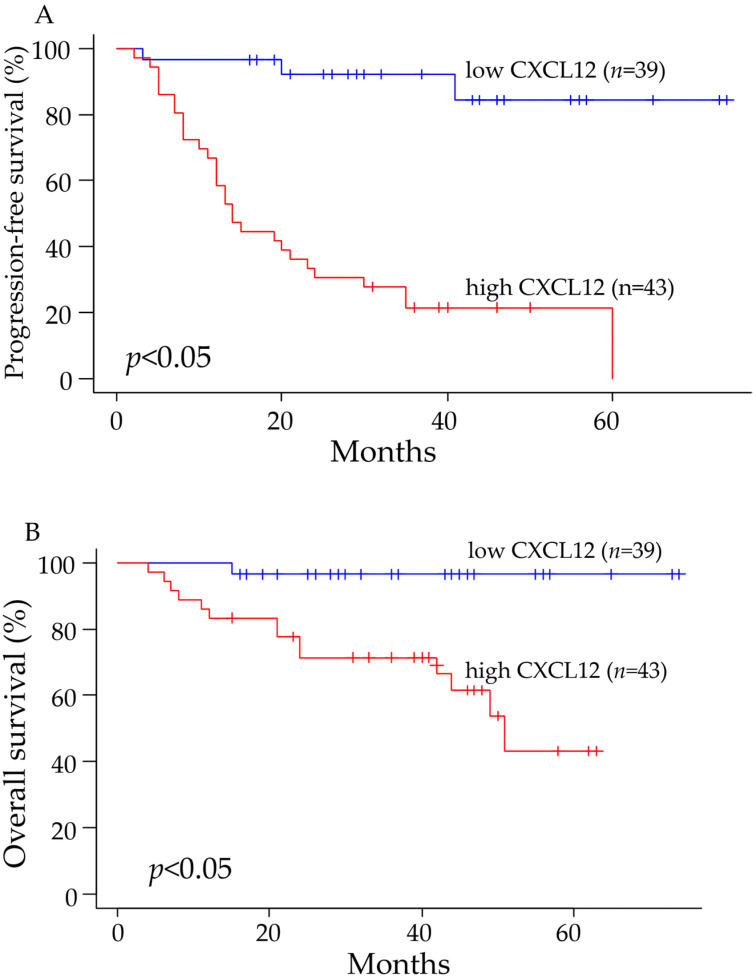
The progression-free survival and overall survival were significantly better in patients with tumors showing low expression of CXCL12. The progression-free survival (**A**) and overall survival (**B**) are illustrated using the Kaplan–Meier method.

**Figure 4:**
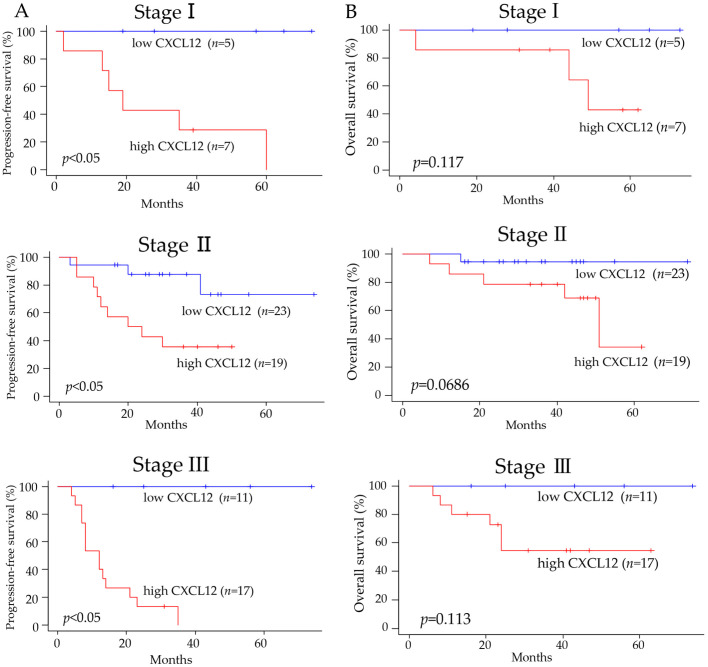
Amelioration of progression-free survival and overall survival in patients with tumors showing low expression of CXCL12 in each clinicopathological stage. (**A**) The progression-free survival was significantly different between patients showing low and high expression of CXCL12 in each pathological stage. (**B**) There was a trend for better overall survival in patients with tumors showing low expression of CXCL12. The Kaplan–Meier method was used to illustrate the data.

Among all subjects, the progression-free survival and overall survival were significantly worse in patients with high expression of CXCL12 compared to patients with low expression (Figure 3A,B). Comparing the progression-free survival of patients at pathological stages I, II, or III showed that the progression-free survival was significantly prolonged in patients with low expression compared to patients with high expression of CXCL12 (Figure 4A). The overall survival was also better in patients with low CXCL12 expression than in patients with high expression at stages I, II, or III, although statistically, no significant difference was observed between the two groups (Figure 4B).

### 2.4. Univariate and Multivariate Analyses

In univariate analysis, the progression-free survival and the overall survival were significantly correlated with a high expression of CXCL12. The overall survival was also significantly correlated with the age of the patients (Table 2). In multivariate analysis, the increased expression level of CXCL12 was a significant predicting factor for progression-free survival and overall survival after adjustment for gender, age, pathological stage, tumor histology, and adjuvant chemotherapy. The patient’s age was also a predictor of overall survival after adjustment for other factors (Table 2).

### 2.5. CXCL12 May Be a Biomarker to Indicate Adjuvant Chemotherapy

**Figure 5:**
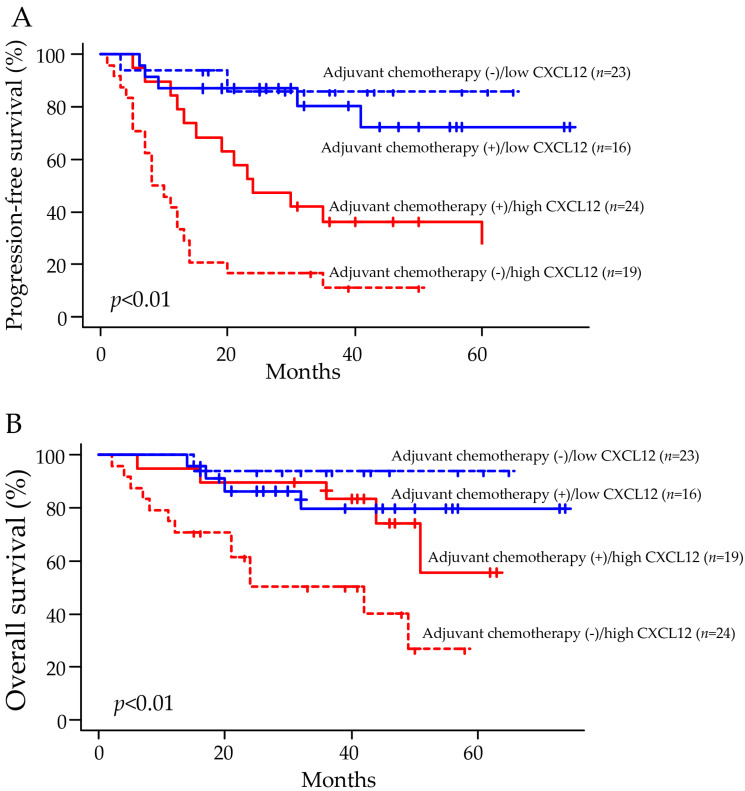
The progression-free survival and overall survival significantly improved in patients with high tumor CXCL12 expression receiving adjuvant chemotherapy. (**A**,**B**) Among patients with tumors showing high CXCL12 expression, the progression-free survival, and overall survival were significantly different between patients with and without adjuvant chemotherapy. Among patients with tumors showing low CXCL12 expression, the progression-free survival and overall survival were not significantly different between patients with and without adjuvant chemotherapy. The Kaplan–Meier method was used to illustrate the data.

Among patients with high CXCL12 expression, progression-free survival and overall survival were significantly better in patients that received postoperative adjuvant chemotherapy than in patients that received no adjuvant chemotherapy. However, among patients with low CXCL12 expression, neither the progression-free survival nor the overall survival was significantly different between patients with and without postoperative adjuvant chemotherapy (Figure 5A,B).

## 3. Discussion

The interaction of chemokines with their receptors plays an increasingly recognized role in the progression of numerous human malignancies [6,11]. The CXCL12/CXCR4 axis is a representative example. The CXCL12/CXCR4 axis is a key mediator of tumor spread, site-specific metastasis, and patient survival [7,8,9]. Previous studies have demonstrated that all major subtypes of NSCLC tumors express CXCR4 and that the main sites of lung cancer metastasis, including bones, liver, adrenal glands, and brain, express high levels of CXCL12 [7,8,11]. Therefore, CXCL12 is considered important in determining the site of metastasis. Phillips and coworkers demonstrated that administering an anti-CXCL12 neutralizing antibody to immunodeficient mice with human NSCLC abrogates organ metastasis [7]. Economidou and associates showed that high CXCL12 levels in the pleural effusion of patients with NSCLC are associated with pleural tumor dissemination [12]. In addition, CXCL12 may also promote the local growth of primary tumors [13,14]. In the present study, we found that a high percentage of NSCLC tumor cells (85.3%) and cancer-associated fibroblasts (65.9%) express CXCL12. Previous evidence suggests that this local production of CXCL12 promotes cancer progression by stimulating tumor cell proliferation, angiogenesis, and epithelial-mesenchymal transition (EMT) [15,16,17].

In this study, we found that NSCLC tumors with low expression of CXCL12 were associated with significantly prolonged progression-free survival compared to tumors with high CXCL12 expression. In addition, multivariate analysis showed that the increased expression level of CXCL12 was a significant predicting factor for progression-free survival and overall survival in NSCLC patients. These findings suggest the potential value of CXCL12 expression by tumor cells and cancer-associated fibroblasts as a marker of prognosis in patients with NSCLC who underwent surgical tumor resection. Another interesting observation in the present study is the association of the tumor CXCL12 expression with response to adjuvant chemotherapy. Among patients with tumors showing high CXCL12 expression, progression-free survival and overall survival were significantly prolonged in patients receiving postoperative adjuvant chemotherapy compared to those that received no adjuvant chemotherapy. However, the progression-free survival and overall survival were not significantly different between patients with and without adjuvant chemotherapy among patients with low CXCL12 expression. These results suggest that adjuvant chemotherapy may benefit patients with tumors showing high CXCL12 expression but not those having low CXCL12 expression.

The study’s retrospective nature, the small number of patients, the single-center design, the variable number of postoperative adjuvant therapy between groups, and the lack of in vitro studies to demonstrate the mechanistic pathways of CXCL12 action in cancer cells (e.g., EMT) are limitations of the present study.

## 4. Materials and Methods

### 4.1. Patients

This study enrolled 82 consecutive patients with NSCLC of real-world clinical practice that underwent complete resection without preoperative chemotherapy or radiotherapy at our institution from 2015 through 2019. Table 1 shows the clinicopathological characteristics of the patients. There were 64 patients with adenocarcinoma, 11 with squamous cell carcinoma, 1 with adenosquamous cell carcinoma, and 5 patients with large cell lung cancer. Twelve patients were in pathological stage I, forty-two were in stage II, and twenty-eight were in stage III. Other genetic abnormalities, including epidermal growth factor receptor mutations, were not evaluated. Informed consent was obtained from each patient regarding clinical records and tissue samples. The Suzuka General Hospital Ethics Committee approved the protocol for this study (No. 271). This study was performed following the Principles of the Declaration of Helsinki.

### 4.2. Immunohistochemistry

All surgical lung specimens were fixed in 10% formalin and then embedded in paraffin. Four-micrometer-thick sections of each sample were prepared for hematoxylin and eosin (H&E) staining and immunohistochemistry using antibodies against α-smooth muscle actin (α-SMA; dilution 1:200; Proteintech, Tokyo, Japan) and CXCL12 (dilution 1:100; Proteintech, Tokyo, Japan). Tumor stromal cells positively stained with anti-α-SMA antibody were considered cancer-associated fibroblasts, as previously described [18]. Staining of α-SMA and CXCL12 was performed following the manufacturer’s instructions. Briefly, primary antibodies were incubated overnight at 4 °C. After incubation with a secondary antibody, immune complexes were detected with 3, 3-diaminobenzidine (DAB) and counterstained with hematoxylin.

### 4.3. Scoring of CXCL12 Tumor Expression

We used the Allred scoring system to assess the expression of CXCL12 in tumor cells and cancer-associated fibroblasts, as previously described [19]. Briefly, the criteria for positive staining were as follows: score 0, negative staining; score 1, <1% of positive cells; score 2, positive cells between 1% and 10%; score 3, positive cells between 11% and 33%; score 4, positive cells between 34% and 66%; and score 5, positive cells between 67% and 100%. The criteria for staining intensity were as follows: score 0, negative; score 1, weak; score 2, moderate; and score 3, strong staining. The Allred score is the sum of the percentage of positive cells and the intensity of staining, and the total score is the sum of the scores for tumor cells and cancer-associated fibroblasts. In the present study, we considered a total Allred score of 0–5 as a low expression, while a total Allred score of >6 was a high expression [20]. All slides were scored blindly by two independent pathologists.

### 4.4. Statistical Analysis

All data were expressed as the means ± standard deviation of the means (SD). The overall survival (OS) and progression-free survival rates after surgical treatment were calculated using the Kaplan–Meier method. The statistical differences between groups were evaluated using the log-rank test. Prognostic factors were assessed by Fisher’s exact test or χ^2^ test. Multivariate analysis was performed using the Cox proportional hazards regression. The hazard ratios were also calculated after adjusting for confounding factors, including age, gender, pathological stage, tumor histology, and adjuvant chemotherapy. Statistical analysis was performed using the R software package version 4.0.3 (R Development Core Team, Vienna, Austria) and the EZR (Easy R) version 1.52 (Saitama Medical Center, Jichi Medical University, Saitama, Japan). EZR is free statistical software that can be downloaded from the following link: https://www.jichi.ac.jp/saitama-sct/SaitamaHP.files/statmedEN.html (accessed on 24 November 2011) [21]. A *p* value < 0.05 was considered statistically significant.

## 5. Conclusions

The results of this study suggest the potential value of tumor CXCL12 expression as a marker to predict prognosis and to indicate adjuvant chemotherapy in NSCLC patients that underwent surgical tumor resection.

## Figures and Tables

**Table 1:** CXCL12 status and clinicopathological parameters in non-small cell lung carcinoma.

Carcinoma					
Overall	Strong	Weak	*p* Value	Expression	Expression
Numbers	82	43	39		
Mean Age	71.5 ± 9.0	72.8 ± 9.2	70.1 ± 8.6	0.169	
Gender					
	Female	26	15	11	0.636
	Male	56	28	28	
Histology					
	Adeno	64	36	28	0.0461
	Squamous	11	2	9	
	Adenosquamous	2	2	0	
	Large	5	3	2	
T Score					
	T1	19	7	12	0.418
	T2	40	22	18	
	T3	19	11	8	
	T4	4	3	1	
N Score					
	N0	28	15	13	0.922
	N1	34	17	17	
	N2	20	11	9	
P-Stage					
	I	12	7	5	0.445
	II	42	19	23	
	III	28	17	11	
Adjuvant Chemotherapy					
	Treated	35	19	16	0.947
	Untreated	47	24	23	
Lymphatic Vessel Invasion					
	Positive	72	38	34	0.869
	Negative	10	5	5	
Blood Vessel					
Invasion					
	Positive	66	36	30	0.437
	Negative	16	7	9	

**Table 2:** Univariate and multivariate analysis.

			Progression-Free Survival			
			Univariate Analysis		Multivariate Analysis	
Factor		*n*	Hazard Ratio (95% CI)	*p* Value	Hazard Ratio (95% CI)	*p* Value
CXCL12	Low	39	Ref		Ref	
	High	43	7.05 (3.09–16.08)	0.0000034	9.99 (4.02–24.84)	0.00000073
Gender	Male	56	Ref		Ref	
	Female	26	0.79 (0.41–1.52)	0.48	0.65 (0.31–1.38)	0.27
Age (years)	<75	50	Ref		Ref	
	≥75	32	1.60 (0.86–2.98)	0.14	0.87 (0.37–2.03)	0.74
Pathological Stage	I or II	54	Ref		Ref	
	III	28	1.66 (0.89–3.1)	0.11	1.88 (0.97–3.66)	0.061
Histology	Non-Squamous	71	Ref		Ref	
	Squamous	11	0.64 (0.23–1.81)	0.4	1.16 (0.38–3.52)	0.8
Adjuvant Chemotherapy	Untreated	47	Ref		Ref	
	Treated	35	0.62 (0.33–1.16)	0.14	0.44 (0.17–1.10)	0.079
			Overall Survival			
			Univariate Analysis		Multivariate Analysis	
Factor		*n*	Hazard Ratio (95% CI)	*p* Value	Hazard Ratio (95% CI)	*p* Value
CXCL12	Low	39	Ref		Ref	
	High	43	3.78 (1.4–10.19)	0.0087	6.88 (2.17–21.85)	0.0011
Gender	Male	56	Ref		Ref	
	Female	26	0.07 (0.01–0.56)	0.011	0.07 (0.01–0.57)	0.013
Age (years)	<75	50	Ref		Ref	
	≥75	32	2.02 (0.89–4.6)	0.092	0.75 (0.25–2.19)	0.6
Pathological Stage	I or II	54	Ref		Ref	
	III	28	1.18 (0.5–2.78)	0.71	1.23 (0.49–3.04)	0.66
Histology	Non-Squamous	71	Ref		Ref	
	Squamous	11	1.45 (0.49–4.27)	0.5	2.57 (0.77–8.56)	0.12
Adjuvant Chemotherapy	Untreated	47	Ref		Ref	
	Treated	35	0.40 (0.16–1)	0.051	0.43 (0.12–1.47)	0.18

## Data Availability

Data is contained within the article.
